# The Your Rheum story: involvement of young people in rheumatology research

**DOI:** 10.1186/s41927-022-00273-z

**Published:** 2022-07-05

**Authors:** Ecem Esen, Suruthi Gnanenthiran, Laura Lunt, Janet E. McDonagh, Your Rheum, Ecem Esen, Suruthi Gnanenthiran

**Affiliations:** 1 Manchester, UK; 2 Department of Chemical and Biological Engineering, University of Sheffield, Sheffield, UK; 3 Centre for MSK Research, University of Manchester, Manchester, UK; 4 NIHR Biomedical Research Centre, Manchester University Hospital NHS Trust, Manchester, UK; 5 Versus Arthritis Centre for Epidemiology, Manchester, UK; 6 Centre for MSK Research, University of Manchester, Manchester, UK; 7 NIHR Biomedical Research Centre, Manchester University Hospital NHS Trust, Manchester, UK; 8 Department of Paediatric and Adolescent Rheumatology, Royal Manchester Children’s Hospital, Manchester University Hospitals NHS Trust, Manchester, UK

**Keywords:** Adolescent, Young adult, Young people, Research involvement, Rheumatology, Research participation, Developmentally appropriate, Peer support

## Abstract

Until recently, young people too often fell into the gaps between services due to restrictive age criteria. Furthermore, their voice was too infrequently heard or was represented by proxies in the form of their caregivers or by adults recalling their youth. The lack of young person involvement in adolescent health research including the arena of paediatric and adolescent chronic disease has been highlighted in current literature. However, the involvement of young people at all stages of health research, from priority setting through to dissemination, is widely advocated. Furthermore, such involvement is considered to be important ethically and, most important of all, has been called for by young people themselves. Young people have clear views about research and these views potentially enhance our understanding of how young people form opinions about research. These opinions in turn informs researchers how to best engage young people (including recruitment and retention) in research. Such involvement of young people ensures that research questions, project methodologies and/or interventions are truly resonant with their lives. This paper describes the development of a national youth advisory group in UK rheumatology, an important addition to the evolving evidence base to support the involvement of young people in rheumatology research. The paper is written with two young people who are members of this group, providing them with an opportunity to learn more about a key component of research—writing papers for publication.

## Background

Until recently, young people too often fell into the gaps between services due to restrictive age criteria, and their voice was too infrequently heard, often represented by proxies in the form of their caregivers and/or by adults recalling their youth. The lack of young person involvement in adolescent health research including the arena of paediatric and adolescent chronic disease has been highlighted [1–3]. Sellars et al. [3] recently reviewed the involvement of youth advisory groups in health research involving 12–18 year olds published in 2019 and less than 1% utilised the advice and expertise of young people during the research. In recent years however, the involvement of young people at all stages of health research, from priority setting right through to dissemination, is widely advocated [4–10]. Such involvement is also considered to be important ethically and, perhaps most important of all, has been called for by young people themselves [11, 12]. Young people have clear views about research [8, 10–13] which in turn enhance our understanding of how young people form opinions about research and thus informs researchers how to best engage young people (including recruitment and retention) in studies. Furthermore, such involvement of young people ensures that research questions, project methodologies and/or interventions are truly resonant with their lives [8–13]. We would now like to add the Your Rheum story to this evolving evidence base to support youth involvement in rheumatology research [11, 12, 14–16].

## Main text

In 2012, the Barbara Ansell National Network for Adolescent Rheumatology BANNAR was established to advocate for research which was both inclusive as well as specifically addressing the adolescent and young adult age groups. As part of this advocacy work, BANNAR aimed to ensure meaningful involvement of young people at all stages of such research. An initial scoping exercise of members revealed that although young people were involved in service development and evaluation initiatives, they were much less involved in research beyond the role of participants. In 2014, research funding was awarded via BANNAR to address this issue and resulted in the largest qualitative nationwide research study to date in this area, involving 63 young people [11, 12, 14] aged 10–24 years. The young people discussed what their research priorities were [11] and went on to identify how they, as young people, wanted to be involved in research in the future [12]. Since then another study from Canada involving 26 young people aged 14–26 years with lupus echoed these results [13].

**Fig. 1:**
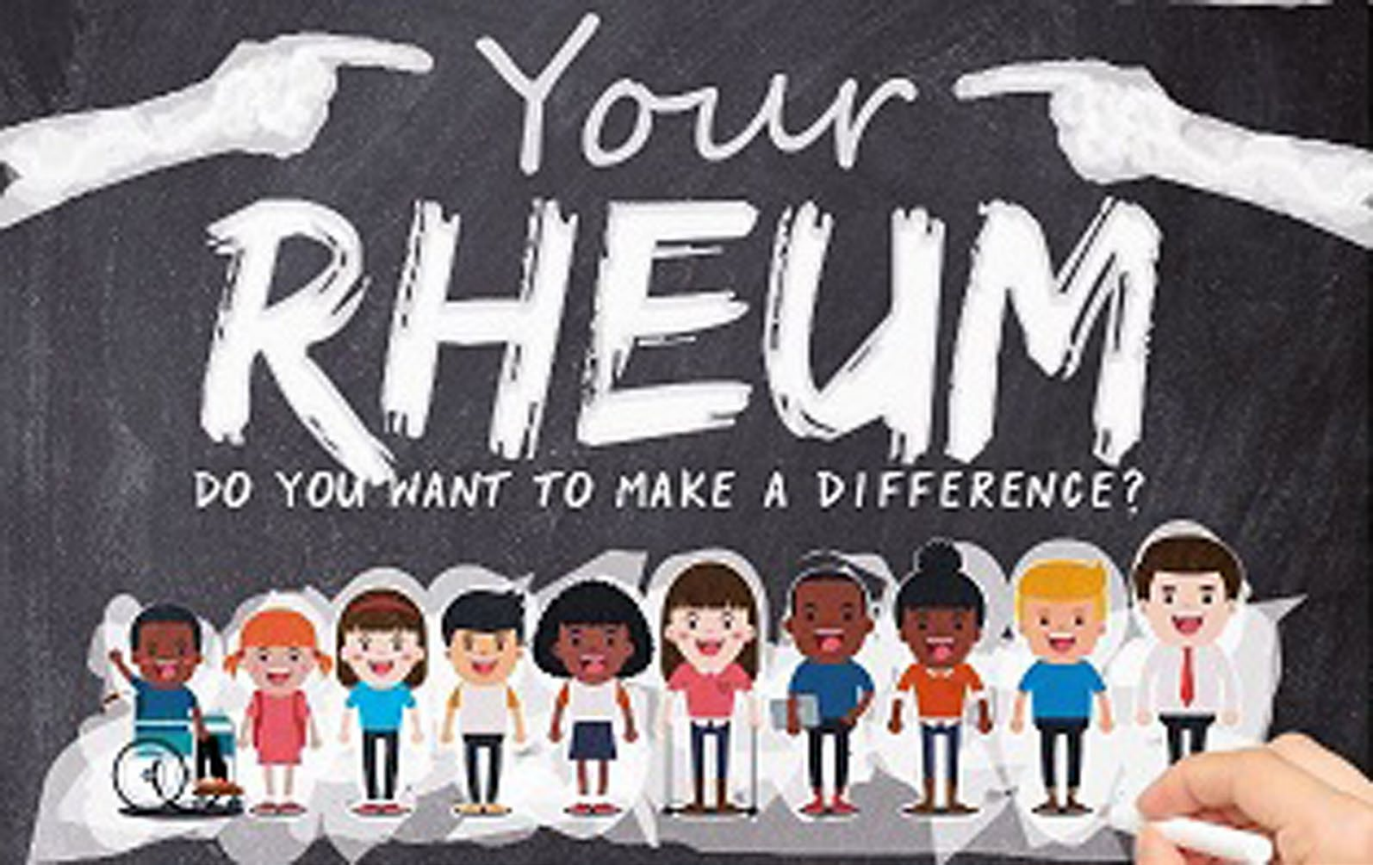
Your Rheum logo

The aforementioned BANNAR research [11, 12, 14] informed the organisation’s involvement strategy, to ensure that as an organisation it would facilitate the ethical and meaningful involvement of young people at all stages of research. This in turn led to Your Rheum being established in 2016 as a national young person ‘research advisory group [15, 16] (Fig. 1).

Your Rheum currently has 45 members from across the 4 nations of the UK with 2 young people working alongside the Your Rheum facilitator (LL) as chair persons, liaising with the professional members of BANNAR. Young people choose which activities to attend and there is no fixed number of meetings members have to attend. They can remain members until they are 25 years if they wish and as they approach this transition, signposting to other opportunities is provided. Opportunity to hear about Your Rheum activities once young people leave is possible by joining the Your Rheum alumni. This flexible approach was adopted in recognition of the challenges young people with relapsing and remitting long term health conditions experience. Also in view of the challenges, navigating adolescence and young adulthood with all the various transitions including educational and health, which this life stage holds in store for them. Prior to the COVID 19 pandemic, the group met twice a year in various geographical locations (frequency limited by funding rather than enthusiasm). During the COVID-19 pandemic the group gained momentum and met virtually every 2 months in addition to a range of online activities which members can do individually. In a recent survey conducted by Your Rheum members presented online at an international rheumatology conference [17], strengths of conducting research involvement activities virtually included logistics and convenience, and for some young people, the development of personal skills such as speaking to others and contributing to group discussions. However, the formal and rigid nature of virtual meetings makes interacting and connecting with other young people difficult. Moving forward, a blended approach to Your Rheum activities is proposed i.e. the inclusion of frequent virtual meetings, as well as regular bi-annual face-to-face meetings (COVID-19 restrictions permitting). However, when planning virtual meetings, consideration to social interactions and opportunities to get to know one another remains important for young people.

At each meeting, key aspects of the research process are specifically highlighted to further members’ understanding of research’. For example, which stage of the research cycle is the particular involvement activity focused upon? A typical meeting will involve 1–2 research projects with the researcher presenting the topic and then an interactive activity to specifically address the area they need input from the young people.

A key lesson from the original research was the importance of feedback—lack of which was described by one young person as “… like taking a test and never getting your grade” [12]. As a result, all researchers accessing Your Rheum are asked to feedback on the progress of their research. Even if funding applications are unsuccessful, it is still important for young people to learn about both the highs and lows of research. All professionals who work with Your Rheum complete an online survey to discuss their experiences of working with the group and provide a future date for a project update. Feedback is continuously monitored with changes/improvements made as/when necessary.

Project updates from professionals are circulated to Your Rheum members via email. Project updates include the following key points; recap of the project and activity with Your Rheum; what the researcher did with Your Rheum’s input (e.g. changes made) and what has happened since Your Rheum’s involvement (e.g. was the project funded). Updates may be in any format e.g. Word document, PowerPoint slide, email, poster or video. Occasionally Your Rheum will receive multiple updates from the professional and/or further opportunities for young people (e.g. writing an abstract). Members have the opportunity to respond, via the Your Rheum facilitator and volunteer to get involved in the research project further, beyond Your Rheum’s facilitation.

To date, Your Rheum has provided input into and helped shape 44 different research projects. How to measure the impact of patient and public involvement in health research continues to be debated with the more frequently reported impacts related to research design and delivery [18]. There are tools reported to support such evaluation e.g. The Public Involvement Impact Assessment Framework Guidance. [19] In Your Rheum we collate simple, descriptive metrics with respect to the 3 key aspects of Your Rheum: the young person (evaluation by the young person and number of young people involved in each activity—male/female); the researcher (feedback as described above) and the research itself (e.g. publications, abstracts, posters, presentations, awards, funding, letters of support).

In agreement with previous authors [4] who have advocated for the importance of a shared learning and development, other key youth development opportunities taken up by the young people include facilitating a session at a national rheumatology conference, presenting at 9 other conferences (7 national, 2 international) in addition to developing a short animation to support recruitment of new members [20]. Your Rheum has also supported members in getting involved in other external activities including: writing book chapters (e.g. [21]) and magazine articles; a public engagement film; photography exhibition; co-presenting at conferences [22], writing conference abstracts [17], and contributing to academic papers such as this one. Your Rheum members have also supported the international campaign—World Young Rheumatic Diseases WORD Day [23] to raise awareness of young people with rheumatic conditions. In addition, Your Rheum enables informal career exploration discussions both between members as well as between members and researchers. Furthermore, the Your Rheum team encourages the work of individual members through supporting/advertising their initiatives and campaigns. For example, one of the Your Rheum members is a founding member of the Learning to Understand Needs and Abilities project LUNA, which aims to support young people with any long-term health condition/disability [24]. Finally, in the original research from which Your Rheum grew, young people have varying opinions regarding payment. Whilst some young people were concerned that such compensation could lead to people becoming involved who did not feel strongly about the research, others felt that such compensation would facilitate a wider range of people getting involved [14]. We therefore offer shopping vouchers for all Your Rheum activities which the majority of young people take up and when meetings are face to face, travel expenses. Young people are also given a certificate once they have completed a Your Rheum activity. This was introduced based on feedback from members.

Your Rheum is currently funded by a Versus Arthritis grant (No: 21593) as detailed in the funding section of the paper. Funding includes salary costs for LL (2 days per week) in addition to thank-you shopping vouchers and travel expenses for the young people. JMcD’s time is unfunded. Most of the research which comes to Your Rheum are one-off activities for grant applications and therefore unfunded and covered by the Your Rheum funding. On occasion, some researchers have a small Patients and Public Involvement PPI budget and can reimburse Your Rheum for the shopping vouchers given to young people for completing the activity. If researchers anticipate several involvement sessions during the course of a funded grant, we request that funding is specifically requested in the grant application for this.

**Fig. 2:**
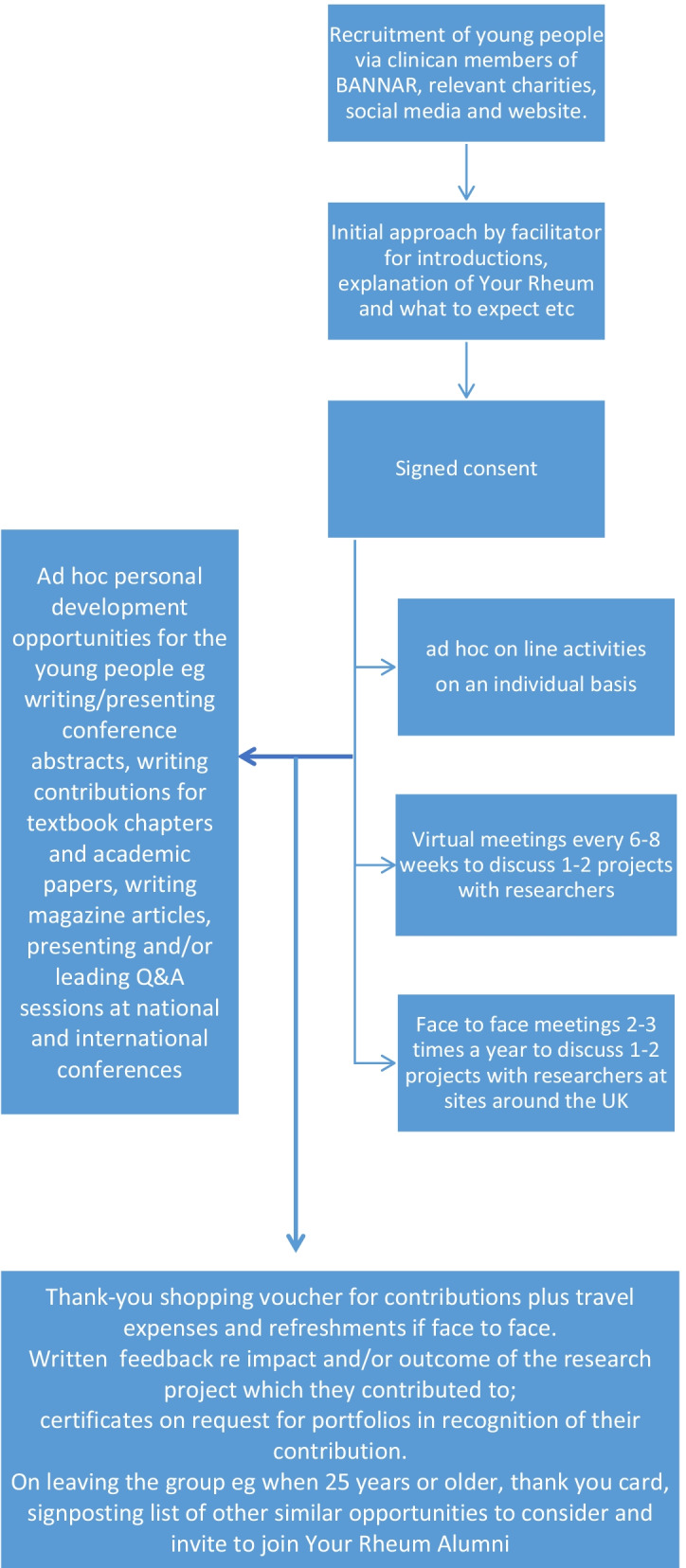
Your Rheum involvement pathway

The involvement pathway is summarized in Fig. 2. Since the creation of the group, the day to day running of the group has essentially remained unchanged other than the move to virtual meetings following the pandemic as we have already described. However the “valued-added” youth development opportunities as described above have evolved in response to the requests and suggestions of the young people themselves. Our hope is that these aspects of the group will continue to evolve over time.

## Your Rheum from the young person perspective

“My name is Ecem and I am 16 years old. I was diagnosed with a rare condition called Behçets disease at age 15. I joined Your Rheum in February 2021 after my doctors and specialist nurses thought I would like to join, as there would be other people my age with similar conditions. I also joined because I thought that it would be great for me to be able to relate to other people living with difficult and challenging conditions.

Your Rheum works on different rheumatology research projects, giving health professionals such as researchers valuable information about young peoples’ health conditions- and also helping us gain a better understanding of research, which helps ensure other people have better care down the line, for example, a quicker diagnosis.

So far, due to the COVID-19 pandemic, I have only been able to join Your Rheum meetings via virtual Zoom calls. This has been a good compromise as they are very accessible. I think that Your Rheum is an amazing way for young people to be able to speak up about important subjects that they believe need more awareness. For example: mental health; raising awareness of young people with long term illnesses; medications and how they can impact on day to day life, (as these can be massively life changing at our age). Most people without a chronic condition do not have to worry about how their health will impact on school work or future aspirations, but we do. Your Rheum meetings are also a great way to be able to speak openly about what affects us on a daily basis, without the fear of being judged. Meetings are a confidential space to speak about issues that other people in the Group may also be going through.

If I had to improve one thing about groups like Your Rheum or offer advice to researchers working with young people, it would be to have more social media presence. Young people use social media as their main communication and information outlet. This is also a good way to raise awareness and helps to remove all the stigma around subjects like chronic health conditions and mental health.

Before Your Rheum I had never been involved in any sort of research. I always knew what the word research meant, but never what it actually entailed. Some of the research done is about the design, and other research is about actually participating. I have also learnt that the process of research is very time consuming and sometimes costly. I have seen that some projects never get approved whereas others could be very popular and are granted funding straight away. However, after joining not only did I participate in the development of research projects as part of the monthly Your Rheum virtual meetings, but I also joined research involvement groups outside of Your Rheum, which has been very insightful.

If I could offer one piece of advice to another young person in a similar position to myself, it would be, speaking out is a very powerful and strong thing to do. From going along to meetings like Your Rheum I have learnt that it is not a bad thing to be able to say what you feel and that this is not a sign of weakness. Your Rheum has helped me gain this confidence, especially whilst voicing my opinion on my care in hospital/medical settings and how my condition affects me.”. Other feedback from other young people are listed in Table 1. All members of Your Rheum were invited (via email) to contribute to the paper by providing written reflections for use in the paper regarding their involvement.

**Table 1:** Feedback from young people in Your Rheum

Your Rheum member	Feedback
1	“During my time in Your Rheum, I have been able to get involved in really cool opportunities such as help with the design of an animated film and work on a research abstract for a PReS [Paediatric Rheumatology European Society] conference. I really enjoyed these opportunities as they allowed me to be involved in exciting work which was very different to anything I had done before, especially the animation. The whole process was really cool and I felt well supported during these activities. Thanks!”
2	“Your Rheum has helped me interact with people who have similar conditions to me. I have had a very positive experience as a Your Rheum member as everyone is very kind and welcoming”
3	“Being part of Your Rheum gives you an insight into current rheumatology research. It helps you become more knowledgeable about your condition and managing it. You might even feel talking to your doctor becomes easier.”
4	“But one of the biggest benefits of being part of Your Rheum, is connecting with other young people like yourself. It’s like a support network. It’s so lovely to share our experiences with one another and not feel alone.”

## Your Rheum from a researcher perspective

Researchers, who work with Your Rheum, are routinely asked to provide written feedback about their experience. This includes how they found planning their activity, (e.g. thinking of questions to ask and activities to engage the young people in conversation); whether they would work with Your Rheum again and if they would recommend working with the group to colleagues. Out of 20 researchers, 17 said they found the initial planning of their activity easy or very easy. One researcher reported it was difficult. However, their additional comments revealed that they found planning their activity alone hard. However, this became easier when they worked with the Your Rheum team. Researchers report that working with the group is valuable to their project. Additional research ideas are generated and/or different perspectives recognised. Often project updates from researchers indicate the impact Your Rheum has had on the project. For example, changes to the research question or reinforcing the importance of the topic all together. All researchers stated that they would work with Your Rheum again and recommended working with the group to their colleagues.

A number of researchers who had recently worked with the group, were contacted and asked to provide a written reflection for this publication. In addition to this, Ecem (with the supervision of the Your Rheum facilitator), conducted a virtual interview with a researcher in order to gather further qualitative data. Ecem selected quotes from the researcher, which she thought were key and wanted to include. These quotes can be found in Table 2.

**Table 2:** Examples of feedback from researchers

Researcher	Feedback
1	“My thoughts 1. Really welcoming, friendly group 2. Incredibly helpful to get young people’s insight and on both occasions, I have come away with something that has led to a change in our research protocol 3. No negative experiences 4. Excellent resource—thank you.”
2	“It was important to incorporate young people as it made my application a lot stronger…Surprised how keen everyone was to get involved…It has given me the confidence to come back to the group moving forward with research, knowing that I will get a good response…Found it very easy to contact the YR facilitator…Received a lot of responses, and really comprehensive responses.”
3	“Working with Your Rheum was a great experience to receive feedback on our proposed project. The members engaged with the tasks and offered us plenty of helpful and insightful feedback, giving points of view we had not considered. Although the meeting was via zoom due to covid times, I was pleasantly surprised with how willing the young people were to share their points of view and debate each other's ideas.”

The Your Rheum team play a vital role in supporting researchers who work with the group, as it is often the researcher’s first time working with young people in this way. The team support the researchers in creating a developmentally appropriate as well as interesting, interactive activity. Effective planning from the outset is key to a positive experience for all concerned including making the activity process clear to the young people and researcher. Clarity of information is important regarding how long an activity will run for, whether reminders will be circulated to members; where the activity will be advertised and/or what the exact role of the researcher will be at a virtual or face-to-face meeting.

## Conclusions

Your Rheum grew from original research with young people to ensure future rheumatology research meaningfully involves young people. To date their involvement has primarily been in the early and formative development stages of research. Our hope is that a future study will be successfully awarded funding, which includes the involvement of Your Rheum throughout the course of the project and hence through to dissemination. It will be important when this happens to further develop our understanding of the complex social processes such prospective involvement reveals for all involved—young people and academics alike—as other studies have revealed [25]. Training of researchers to ensure meaningful involvement of young people will be important moving forward. This includes mechanisms for feedback and appropriate reference to their involvement in publications arising from the research, so that future literature reviews will demonstrate improvement, compared to that of Sellars’ et al. findings [3]. Finally, as Ecem so clearly described in her section, we should never underestimate the peer support aspect of such groups and the value young people place on this as a reason for their involvement.

## Data Availability

Not applicable.

## Abbreviations

BANNAR: Barbara Ansell National Network for Adolescent Rheumatology
WORD: World Young Rheumatic Diseases Day
LUNA Project: The Learning to Understand Needs and Abilities project
PReS: Paediatric Rheumatology European Society
